# Early developmental assessment with a short screening test, the STEP, predicts one-year outcomes

**DOI:** 10.1038/s41372-018-0234-4

**Published:** 2018-10-09

**Authors:** Laurel Gower, Dorothea Jenkins, Jamie L. Fraser, Viswanathan Ramakrishnan, Patty Coker-Bolt

**Affiliations:** 1 College of Medicine, MUSC, Charleston, SC USA; 2 Department of Pediatrics, MUSC, Charleston, SC USA; 3 Rare Disease Institute, Children’s National Medical Center, Washington, D.C. USA; 4 Department of Public Health Sciences, MUSC, Charleston, SC USA; 5 Division of Occupational Therapy, College of Health Professions, Charleston, SC USA

**Keywords:** Physical examination, Paediatrics

## Abstract

**Objective:**

To evaluate the Specific Test of Early Infant Motor Performance (STEP), a rapid screening test of preterm infants at risk for developmental delay.

**Study Design:**

We prospectively studied 23 preterm infants’ performance on the STEP and the Test of Infant Motor Performance (TIMP) at term and 3 months, and on the Bayley-III at 12 months. We investigated the psychometric qualities of the STEP and determined STEP cutoff scores for low and high-performing infants.

**Results:**

STEP scores at term and 3 months strongly correlate with 12-month Bayley-III gross motor and cognitive scaled scores, while TIMP scores did not. The STEP showed excellent reliability and required 6–10 min to administer.

**Conclusion:**

STEP is a short, easy to administer, early developmental assessment with unique scoring that emphasizes qualitative and quantitative aspects of muscle tone in movements and predicts 12-month Bayley gross motor and cognitive scaled scores.

## Introduction

Premature birth is a major risk factor for developmental delays, which may result in a diagnosis of cerebral palsy (CP) at 18–24 months of age or cognitive deficits [1, 2]. Eligibility for early intervention monitoring varies by state, but in general infants must demonstrate significant milestone delays in order to be referred to physical or occupational therapy within the first 12 months. Even for preterm infants who are followed every 3 months in high-risk clinics, the central tenet of developmental assessment is to wait until abnormalities manifest before referring for treatment. With later initiation of treatment, a significant window of neuroplasticity for preterm infants may be missed, in which targeted therapy could prevent negative neurological outcomes.

Earlier and more frequent screening using a test of developmental skills could potentially address the well-documented delay in referral and begin to change the orientation to prevention instead of post hoc treatment. However, to change the paradigm of referral later in infancy to early identification and treatment, we need a rapid, reliable, early screening test for global developmental problems. Existing comprehensive developmental tests have low compliance rates (23%) among general pediatricians [3], including the validated Test of Infant Motor Performance (TIMP) and Bayley Scales of Infant and Toddler Development (Bayley-III). The TIMP is a 42-item motor development test that takes ~45 min to administer at 34 weeks to 4 months corrected gestational age (CGA). The Bayley-III is a comprehensive test that assesses language, cognition, fine motor, and gross motor development and takes 60–90 min to administer, depending on the age and ability level of the child. Many physicians cite time constraints, lack of specialized training for test administration, and lack of reimbursement for time as the main reasons for foregoing standardized screening [3]. Additionally, currently used screening tests involve prolonged handling that  can be detrimental to fragile neonates [4]. Compared to their full-term counterparts, preterm infants are especially vulnerable to the stressful physiological effects of neurodevelopmental testing [5].

The Specific Test of Early Infant Motor Performance (STEP) is a novel developmental screening test for preterm infants designed for rapid and early detection of motor problems. In constructing this test, we previously performed Rasch partial credit analysis of all 42 items in the TIMP combined with kinematic study of early infant motor patterns, to select 10 movements that showed the greatest discrimination between preterm infants of different motor abilities [6]. These movements reflect anti-gravity flexion and extension of the head and neck, movement in the arms and legs, and tone in the shoulder girdle and pelvis, which are foundational for high level motor movements in the first year of life. We also previously investigated motion kinematics for three of the STEP items, and correlated these angles and item scores with neuroimaging [7–9]. We established new scoring scales for each item, tested the scoring scales and items in a factor analysis, and piloted version 3 of the STEP in the clinic for feedback on the representation of the pictorial scoring scales, and to obtain data on clinical utility. Finally, we enrolled a new cohort of preterm infants in a prospective study. Our premise is that a well devised and validated early motor screening test could be administered in the nursery before discharge and in the clinic at term and 3 months, and predict later developmental outcomes.

With these studies, we determined the psychometric properties of this new early neurodevelopmental assessment (STEP), and prospectively assessed the predictive value of the STEP’s novel scoring scale for 12-month outcomes by Bayley-III assessment, compared with concurrent scores on the gold standard TIMP. We determined STEP cutoff scores for high and low-performing infants based on Bayley III motor scores at 12 months. We hypothesized that the STEP and TIMP would be positively related to motor outcomes at 12 months CGA.

## Methods

### Determining psychometric properties of the Specific Test of Early Infant Motor Performance (STEP)

#### Specific STEP Items

**Fig. 1:**
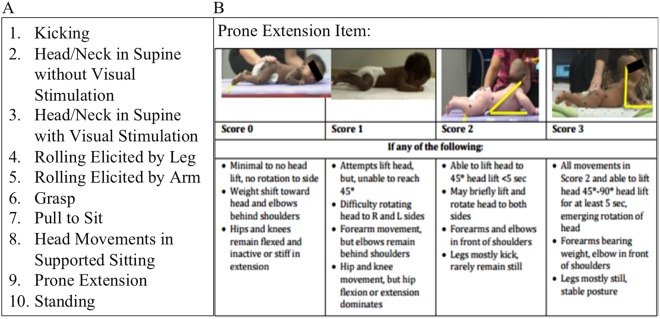
**a** Ten items of the Specific Test of Early Infant Motor Performance (STEP). **b** Example of STEP pictorial scale for one item, Prone Extension

The ten STEP items comprise pull to sit, prone extension, head movement in supine with and without visual stimulation, head movements in supported sitting, supported standing, grasp, rolling elicited by leg and arm, and kicking (Fig. 1 and Supplemental Figure 4). These are elicited by a therapist with the infant in different positions to assess overall posture and the quality of movement of the head, arms, and legs as well as visual grasp responses while the infant is supine. A unique feature of the STEP is that each item looks at the quality of movement, not just presence or absence of the motor skill. For example, the Prone Extension item rates an infant on how high the infant can lift and hold the head up while prone, as well as the position and movement of upper and lower extremities, all of which are required for successful head lift (Fig. 1).

#### STEP scoring procedures

Each of the 10 items is rated on a unique 4-point pictorial scale accompanied by brief descriptions, with scores from zero to three, progressing from immature to more mature motor movements, and summed for a maximum total score of 30 (presented in Fig. 1).

The unique STEP scoring scale was derived from careful review and kinematic analysis of existing video recordings of a prior preterm cohort, in devising a novel linear scale (0–3 points) for each of the STEP items [7, 8]. A consensus panel of pediatric specialists included one PhD occupational therapist, three clinical occupational therapists, two developmental pediatricians, and one developmental pediatric geneticist, who reviewed and revised the new scales, providing feedback on each item of the STEP. Pictorial representations were refined based on this feedback.

#### Exploratory factor analysis

Each item on the STEP encompasses functional coordination of a variety of muscle groups as well as intrinsic tone of the muscles, and the latent constructs of each movement cannot be directly assessed. Therefore, to determine the underlying constructs of the STEP items and to better define its properties, we next performed an exploratory factor analysis. STEP scores were derived from the videotapes of motor testing on the 22 preterm infants previously described, 9 of whom were low performing on TIMP at 3 months and 13 who were high performing [7]. Nine STEP items were able to be scored from these videos and were analyzed for weighting in 3 motor and tone constructs: *head control*, *upper extremity tone and movement*, and *lower extremity tone and movement*. Factor loadings vary from −1 to 1, indicating the strength and direction of the relationship between the items and the latent constructs. We also determined the percent of variance in the nine-item STEP score explained by each of these constructs to further characterize the STEP test and what it measures.

#### Reproducibility and clinical utility

We determined the intra- and inter-rater reliability of the STEP assessment when scored by expert and novice raters. We provided a brief 15-min training session, which included explanation of STEP scales to novice raters, who then watched and scored videotaped assessments using the STEP. Three experienced and five novice raters observed and scored five video assessments of both high- and low-risk infants, repeating the process with same video clip one-week later. Expert raters were an occupational therapist (OT) and OT students who developed and refined STEP scales. Novice raters included a research assistant and neonatal providers (physician, hospital OTs) who were unversed in the STEP assessment. Intra-class correlation coefficient (ICC) was calculated using a 95% confidence interval.

Clinician raters gave feedback on clarity, practicality, relevance, and importance of the STEP's clinical utility. The STEP was evaluated in the nursery before discharge and during routine visits for preterm infants from term to 3 months CGA in high-risk clinic, for ease of use by novice (pediatric residents) and experienced (neonatologist, developmental pediatrician, occupational therapist, neonatal nurse practitioner) infant raters (*n* = 8 infants, *n* = 5 practitioners). Quantitative and qualitative markers of clinical utility were recorded during the visit: time efficiency; ease of interpretation of the scales and scoring by attending and residents; flow and ordering of test items; and direct feedback from pediatric clinicians who administered the STEP.

#### Differences in STEP and TIMP scoring

We performed quadrant analysis of total STEP versus total TIMP scores of individual infants to visually demonstrate the differences in scoring scales. We also compared quadrant analyses of scores for individual infant’s performance on STEP item with the corresponding item on the TIMP. We evaluated the change in individual item score over the term to 3 months period by the STEP and TIMP scoring scales to demonstrate differences in scoring/evaluation of the movement.

#### Prospective evaluative validity of STEP

The prospective study was approved by the MUSC Institutional Review Board (IRB), and we obtained consent from parents in the neonatal intensive care unit, including a consent to photograph and videotape participants. Inclusion and exclusion criteria were same as the previous cohort. We enrolled 30 preterm infants born between 24 and 34 weeks gestational age (GA) and discharged by 44 weeks GA, and excluded infants with congenital brain malformations or other major congenital anomalies to reduce heterogeneity in the sample, and because these conditions might result in no substantive developmental progress over 3 months. Assessments were performed at term (37–42 weeks GA), and at 3 and 12 months CGA.

These time points were chosen to capture the significant changes in movement and tone over this three month period, remain within the valid range of the TIMP (concurrent gold-standard), and ultimately allow identification of preterm infants early after discharge to facilitate early referral to therapeutic services. All references to 3 and 12 months of age represent CGA. The STEP and TIMP were administered in the same session, in the same order, at term and 3 months by the same occupational therapist (OT). For safety reasons, the OT was aware of the infants’ medical status. The STEP was performed first and then followed by the TIMP assessment. The examiner provided breaks as needed if the infant showed signs of fatigue, distress, or hunger. Each session typically lasted 45-min to 1-h depending on need for brief breaks. The Bayley-III was administered in a separate session at 12 months to the 19 infants who returned for follow-up, by an OT research assistant who was certified in Bayley administration and blind to previous test scores, and lasted approximately one and a half hours.

### Test of infant motor performance (TIMP)

TIMP is a valid and reliable assessment and the only tool to demonstrate adequate evaluative validity for premature infants from 34 weeks through 4 months CGA [10–13]. The TIMP has convergent validity with the Bayley-III motor scales administered at 6 months, but has not been tested against 12-month Bayley-III scores [14]. The standardized TIMP consists of 42 items and requires 45 min to administer by trained personnel. Norm referenced cutoff scores for below average infants are ≤45 at term and <89 at 3 months, or two standard deviations below the mean [15].

### The Bayley-III

The Bayley-III is considered the gold-standard neurodevelopmental assessment for infants age 1–42 months at risk for motor, language, or cognitive delays [15–17]. Toddlers are traditionally assessed for cerebral palsy at 18–24 months of age, but 12 months is a typical period for diagnosis of developmental delays. Below normal or low Bayley-III gross motor scaled scores were defined as <9, 1 standard deviation below the mean [16, 17]. The domains measured and reported were cognitive, language, fine motor, and gross motor.

### STEP cutoff scores

Receiver operating characteristic (ROC) curves were created in order to determine STEP cutoff scores at term and 3 months that distinguished high from low-performing infants. The STEP score at term or 3 months served as the continuous variable, and the Bayley-III gross motor scaled score at 12 months as the dichotomous variable in the ROC curve (low score <9/high score ≥ 9) [16–18]. Analysis of the ROC curve for AUC and 95% CI was performed to determine the STEP values at term and 3 months with optimal sensitivity and specificity for gross motor scores at 12 months. These cutoff scores were then used to compare how infants were characterized as either low or high performing based on the STEP and TIMP performed at the same session at term and 3 months, using *Χ*^2^ test.

### Statistical Analysis

SPSS software (IBM, version 23) was used to analyze relationships between STEP scores, Bayley composite and scaled scores and TIMP scores using Spearman’s rho for nonparametric variables. We used chi squared, sensitivity, and specificity analyses to compare infants with high and low-performing STEP scores against infants with concurrent high and low TIMP scores, using standardized TIMP cutoff scores [Infant Motor Performance Scales, LLC]. We used these same analyses to compare infants with high and low-performing TIMP scores with normal and low normal Bayley-III gross motor scores. Correcting for multiple comparisons using the bonferroni approach for the STEP scores with 3 outcomes (concurrent TIMP, Bayley motor and cognitive scores), significance is designated at *p* ≤ 0.017. . We also calculated positive and negative likelihood ratios and posttest probabilities for STEP score predicting below/ normal Bayley scores.

## Results

### Evaluation of the latent constructs of the STEP

The exploratory factor analysis comprised nine STEP items, excluding rolling with arm which we could not accurately score from the existing videotapes [7]. The nine movement and tone items of STEP were grouped into three meaningful latent constructs of head control, and upper and lower extremity tone and movement (Supplementary Figure 5). Overall, the latent construct of ‘*Head control*’ contributes 68% of the STEP variance, while *‘Upper and lower extremity tone and movement’* combined contribute 22 and 12%, respectively, of the variance of STEP scoring.

### Reliability and clinical utility of the STEP

Intra- & inter-rater reliability for expert raters were excellent, and for novice raters was good to excellent (Table 1). Out of 60 item score comparisons, the mean difference between the expert and novice rater was 0 (standard deviation = 0.41). Fifty item scores showed no difference in item score between raters, while 10 scores differed by + or −1. The time required for administration of the STEP in typical nursery and high-risk clinic settings with experienced and novice raters (*n* = 7) was on average 6 min (up to a maximum of 10 min for a novice rater), compared with reported 10–30 min for the Alberta Infant Motor Scale (AIMS) and  45 min for the TIMP [15]. Qualitative feedback indicated that the test “fit well into the normal routine of the clinic”, it was “fast, easy and intuitive”, “scoring became easier and faster with practice”, and “identifies motor behaviors indicative of potential delays”.

**Table 1:** Intra- & inter-rater reliability for expert raters and novice raters

	Expert raters	Novice raters
Intra-rater reliability	ICC = 0.92–0.96	ICC = 0.82–0.92
Inter-rater reliability	ICC = 0.91–0.95	ICC = 0.84–0.94

### Demographics of the cohort for prospective predictive value

In the new cohort of 30 preterm infants enrolled to determine predictive validity and cutoff scores, twenty-three infants completed early developmental testing by returning for STEP and TIMP tests at term and 3 months (*n* = 22 at each time point; 2 infants returned for only the term or 3 month assessment, Table 2). In this relatively healthy cohort, three infants had intraventricular hemorrhage (IVH) grades 1–3 diagnosed by routine head ultrasound. No infant had grade 4 IVH. The infant with grade 1 IVH scored below average on two of five motor indices (12-month Bayley-III gross motor and 3 month STEP), and the infants with grade 2–3 IVH scored below average on all motor indices. Two infants had small areas of periventricular leukomalacia (PVL): One infant had bilateral PVL, hemiplegic CP, and below average scores on Bayley motor subscales at 12 months; The other infant had right-sided PVL and scored average on motor subscales at 12 months. Clinical sepsis was diagnosed in 6 infants, including all infants with bilateral PVL and IVH. Two infants changed from low to high-performing category on the STEP between term and 3 months, while no infant changed from high to low performance on STEP between term and 3 months. The one infant who was only assessed at term was high performing, and one infant who was only assessed at 3 months was low performing. Nineteen infants returned for the Bayley-III testing at 12 months.

**Table 2:** Patient Demographics

	Number
Total Subjects	23
Gender: Male	12
Female	11
Race: African-American	6
Caucasian	17 (1 Hispanic)
GA at birth	28.75 ± 3.37 wks
GA at MRI Scan	41.94 ± 1.53 wks
BW grams	1235 ± 508.5
STEP term:	
Low performance (≤16)	10
High performance ( > 16)	12
STEP 3 mo:	
Low Performance (≤22)	9
High Performance (>22)	13
TIMP term:	
Below average (≤45)	11
Average (>45)	11
TIMP 3 mo:	
Below Average (<89)	5
Average (≥89)	17
Bayley GM SS^a^:	
Below Average (<9)	8
Average ( ≥ 9)	11
Sepsis	4
Chorioamnionitis	3
IVH Grade 1	1
IVH Grade 2	1
IVH Grade 3	1
PVL	2

*GA* gestational age, *BW* birthweight, *Bayley GM* *SS* Bayley-III Gross Motor scaled score, *IVH* intraventricular hemorrhage, *PVL* periventricular leukomalacia

^a^No infants scored below average on Bayley-III cognitive scaled score at 12 months

### STEP scores correlate with TIMP scores

**Fig. 2:**
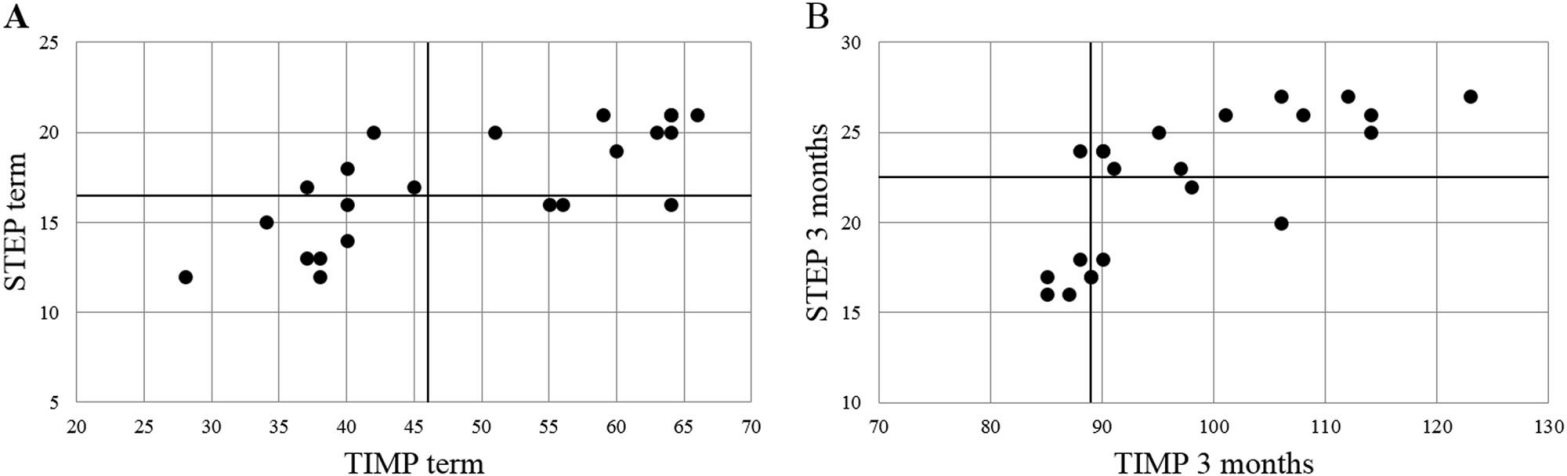
Scatter plots of STEP and TIMP scores from individual preterm infants (**a**) at term and (**b**) at 3 months CGA. Cutoff scores for high and low-performing infants for each test are noted by the cross bars (STEP ≤ 16 and TIMP ≤ 45 at term; STEP ≤ 22 and TIMP < 89 at 3 mo CGA). Individual infants characterized as high-performing by both tests are in the right upper quadrant, and low-performing infants are in the left lower quadrants

To determine how closely the STEP tracked the longer TIMP assessment, we compared individual infant’s total TIMP and STEP scores at term and 3 months in a quadrant analysis (Fig. 2a, b). STEP scores were significantly, but not precisely, correlated with concurrent TIMP scores both by quadrant analysis and spearman’s rho at term (*r*_s_ = 0.762, *p* = 0.000038, *n* = 22) and at 3 months (*r*_s_ = 0.820, *p* = 0.000003, *n* = 22).

### STEP at term and 3 months predicts Bayley-III motor and cognitive scores at 12 months

**Fig. 3:**
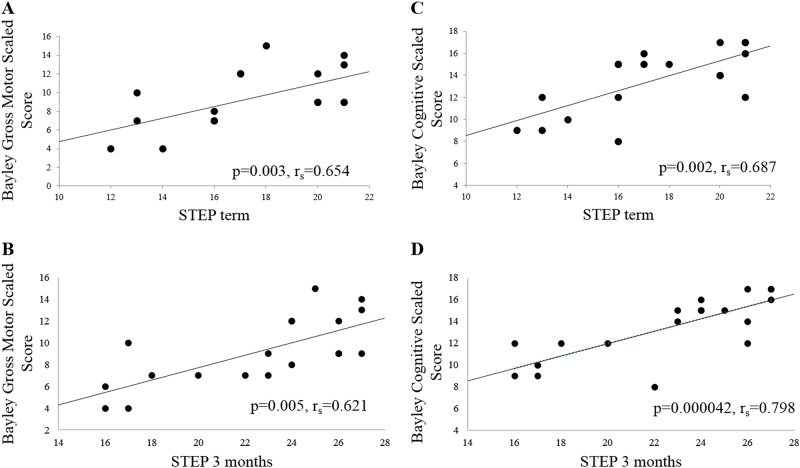
**a** Bayley GM scaled score at 12 months CGA are correlated with STEP at term (*p* = 0.003, *r*_s_ = 0.654, *n* = 18). **b** Bayley GM is correlated with STEP at 3 months (*p* = 0.005, *r*_s_ = 0.621, *n* = 19). **c** Bayley cognitive scaled score is correlated with STEP at term (*p* = 0.002, *r*_s_ = 0.687, *n* = 18). **d** Bayley cognitive is correlated with STEP at 3 months (*p* = 0.000042, *r*_s_ = 0.798, *n* = 19). Overlapping points due to repeated score combinations account for decreased number of data points relative to sample size

There is a significant correlation between STEP scores at term and Bayley-III gross motor scaled score at 12 months (*r*_s_ = 0.654, *p* = 0.003, *n* = 18, Fig. 3a). STEP scores at 3 months also significantly correlated with Bayley-III gross motor scaled scores at 12 months (*r*_s_ = 0.621, *p* = 0.005, *n* = 19, Fig. 3b).

A significant association exists between STEP scores and Bayley-III cognitive scaled scores. STEP at term positively correlates with Bayley-III cognitive scaled scores at 12 months (*r*_s_ = 0.687, *p* = 0.002, *n* = 18, Fig. 3c). STEP at 3 months also positively correlates with Bayley-III cognitive scores at 12 months (*r*_s_ = 0.798, *p* = 0.000042, *n* = 19, Fig. 3d). All correlations of the STEP scores with other outcome assessments were still significant when corrected for multiple comparisons.

### STEP cutoff scores

Analysis of the ROC curve using the 12-month Bayley III gross motor scaled scores revealed a clear dichotomy between infants with normal and low to below normal performance on the Bayley at 12 months, and their corresponding STEP scores at term (AUC = 0.929, 0.790–1.000 95% CI), with a STEP cutoff of ≤ 16 at term giving optimal sensitivity and specificity to predict Bayley gross motor performance (Sn = 1.00, Sp = 0.909). A similar dichotomy existed when relating high and low Bayley performance with their corresponding STEP scores at 3 months (AUC = 0.909, 0.772-1.000 95% CI). ROC analysis yielded a STEP cutoff of ≤ 22 at 3 months for maximum sensitivity and specificity to predict Bayley gross motor performance at 12 months (Sn = 0.75, Sp = 0.909).

The positive likelihood ratio (LR) for Bayley gross motor score < 9 at 12 months with a STEP score ≤ 16 at term is 11, for a posttest odds of 7, and posttest probability of 87.5%. The negative LR for Bayley gross motor score < 9 at 12 months with a STEP score > 16 at term is 0. The positive LR for Bayley gross motor score < 9 at 12 months when the STEP score ≤ 22 at 3 months is 8.25, for a posttest odds of 6, and posttest probability of 85.7%. The negative LR for Bayley gross motor score < 9 at 12 months when the STEP score ≥ 22 at 3 months is 0.275, for a posttest odds of 0.2, and posttest probability of 16.7%.

When used to predict average or below average performance on TIMP, STEP cutoff of ≤ 16 at term yielded a sensitivity of 0.636, specificity of 0.727, negative predictive value of 0.667, and positive predictive value of 0.700. STEP 3 month cutoff of ≤ 22 gave sensitivity of 0.800, specificity of 0.706, negative predictive value of 0.923, and positive predictive value of 0.444 for TIMP scores at 3 months. Using these cutoffs, chi squared test showed that high and low performance on STEP was not associated with high and low performance on TIMP at term (*χ*^2^ = (1, *n* = 22) 2.933, *p* = 0.087) or at 3 months (*χ*^2^ = (1, *n* = 22) 4.090, *p* = 0.043) after corrections for multiple comparisons.

### TIMP does not correlate with Bayley

Surprisingly, TIMP scores at term and 3 months did not correlate with Bayley-III gross motor scaled scores at 12 months in our cohort using Spearman’s rho (term: *r*_s_ = 0.125, *p* = 0.621, *n* = 18; 3 months: *r*_s_ = 0.418, *p* = 0.075, *n* = 19). TIMP scores also did not correlate with Bayley-III cognitive scaled scores at 12 months (term: *r*_s_ = 0.237, *p* = 0.343, *n* = 18; 3 months: *r*_s_ = 0.451, *p* = 0.053, *n* = 19). When TIMP scores were used to predict average and below average scores on Bayley-III gross motor, TIMP cutoff of < 45 at term had a sensitivity of 0.572, specificity of 0.545, negative predictive value of 0.667, and positive predictive value of 0.444. TIMP cutoff of <89 at 3 months had a sensitivity of 0.333, specificity of 0.909, negative predictive value of 0.667, and positive predictive value of 0.750. Chi squared test showed that normal and low-below normal performance on the Bayley-III gross motor test was not associated with high and low TIMP performances at term (*χ*^2^ = (1, *n* = 18) 0.234, *p* = 0.629) or 3 months (*χ*^2^ = (1, *n* = 19) 2.249, *p* = 0.134).

## Discussion

This report explores the associations between the novel assessment, the STEP, a rapid, early developmental screening of the preterm infant, and gold-standard developmental assessments. Our data indicate that the STEP scores at term and 3 months CGA may adequately predict longer-term motor development. We demonstrate that a STEP cutoff of ≤16 at term and ≤22 at 3 months differentiates gross motor performance on the Bayley-III at 12 months with excellent sensitivity and specificity by ROC curves. These STEP cutoff scores were also associated with concurrent TIMP scores at term and at 3 months.

TIMP in general showed poor to moderate predictive ability for Bayley gross motor scores at 12 months in our cohort. A prior study correlated Bayley-III at six months and the TIMP administered between 33 and 39 weeks CGA with sensitivity of 0.86 and specificity of 0.68 [14, 19]. TIMP has not shown predictive value against the Bayley at 12 months or later time points [14]. In our cohort, STEP scores at term and 3 months CGA had a stronger predictive correlation with 12-month Bayley gross motor scaled scores, with much better sensitivity and specificity than TIMP scores.

We also demonstrate that the STEP takes approximately 10 min to administer and score, and can be quickly learned by a variety of neonatal care providers, who demonstrate good inter- and intra-rater reliability. The STEP works well in clinic from a practical standpoint of patient flow and provides a quantitative assessment of tone and movements which allows developmental tracking with a short screening test. The STEP provides an opportunity to recognize early abnormalities of tone and movement patterns in a standardized test and refer for therapy before milestones are delayed or abnormal movements become fixed.

Although there are other validated tests that may screen for motor deficits in the nursery and in the clinic, most are lengthy exams that involve prolonged handling of the infant. The General Movement Assessment (GMA) is one of the few validated tests that is short and does not involve any handling. The GMA analyzes an infant’s spontaneous movements and predicts cerebral palsy at 2 years of age with high sensitivity and specificity (Sn: 0.98, Sp: 0.91) [20]. The GMA is a videotaped assessment, but multi-day training or off-site processing is required for reliable scoring. It is valid from term to 20 weeks CGA. While the GMA is valid for predicting CP, it has variable sensitivity and specificity in predicting less severe motor outcomes at 2 and 4 years (Sn range: 0.50–1.00, Sp range: 0.42–0.88) [21].

Recent studies also advocate using a combination of assessment tools during the first year of life [22, 23]. The rate of false positives when administering one assessment is common, and follow-up of infants at high risk of impairment at more than one-time point with a combination of assessment tools may be of benefit [23, 24]. While the GMA may be used to accurately diagnose CP, the STEP may be useful to identify infants at risk for both cognitive and motor developmental delays. After further validation, the STEP and the GMA may complement each other and synergistically broaden clinical prognostication in early development [22].

The limitations of this study include the number of subjects and the 12-month assessment of outcomes. We chose twelve months as a typical period for diagnosis of developmental delays, not for the definitive diagnosis of cerebral palsy. Also 12-24 month cognitive scores on the Bayley II and III may relate significantly to later cognitive outcomes at 4–5 years, but diagnosis of cognitive impairment as well as cerebral palsy must be assessed at later time points [25, 26]. While much effort has gone into early prediction of cerebral palsy, less severe or global delays could also benefit from early referral and therapy. The STEP could be used to quantify early movement problems in high-risk infants, refer these infants earlier and then track response to targeted interventions.

The STEP needs to be validated in a larger cohort, including those with more obvious brain injury or complicated neonatal courses. However, these data indicate the STEP may identify infants at risk for delays, which would allow earlier initiation of targeted therapy. With a validated early screening test, the concept of preventative developmental therapy could move closer to reality. In our cohort, STEP scores at term are strongly associated with future motor and cognitive scores on Bayley-III. Ultimately, the STEP could be implemented before discharge in the nursery and in follow-up clinics as a screening test for early intervention, and potentially improve long-term outcomes. By integrating the STEP developmental assessment tool into clinical practice, neonatal intensivists, primary care pediatricians, and early intervention therapists could one day better determine which at-risk infant needs additional proactive rehabilitative services based on an objective metric with good predictability.

## Electronic supplementary material

Supplemental Figure 4

Supplemental Figure 5
