# Supplementary material for: Early developmental assessment with a short screening test, the STEP, predicts one-year outcomes
Source: J Perinatol. 2018 Oct 9;39(2):184–92. doi: 10.1038/s41372-018-0234-4 (PMC6349683; doi:10.1038/s41372-018-0234-4)
Supplement: Supplementary file 1 — Supplemental Figure 4 [file 41372_2018_234_MOESM1_ESM.pdf]

Specific Test of Early Infant Motor Performance (STEP) *Version 3.0*

Infant ____

Test Date (Y/M/D)____________________________________

Birth Date (Y/M/D)____________________________________

Current Age (Mo/Days)_________________________________

Mo/Days Preterm _____________________________________

Adjusted Age (Weeks/days)_____________________________

Examiner's name_________________________________

TERM cut-off score: TOTAL STEP score ≤ 16 is considered high risk for later developmental delay

☐ High Risk ☐ Low Risk

3-Month cut-off score: TOTAL score ≤ 22 is considered high risk for later developmental delay

☐ High Risk ☐ Low Risk

Summary Score Sheet

**Score**	**Item**
____	Kicking
____	Head/Neck in Supine without Stimulation
____	Head/Neck in Supine with Visual/Auditory Stimulation
____	Rolling Elicited by LEG
____	Rolling Elicited by ARM
____	Grasp
____	Pull to Sit
____	Head Movements in Supported Sitting
____	Prone
____	Standing
_____	**TOTAL SCORE**

Examiner Instructions:

- Please complete all 10 items of the STEP. After each item, rate the infant's motor performance using the scale provided.

Glossary of Terms:

Flexor Recoil: Legs spring back into the flexed position after being flexed up to abdomen by examiner then quickly extended and released
Reciprocal Kicking: Spontaneous alternating kicking of both legs
Lateral Neck Righting: Active lateral flexion of the neck (side bending) when infant is in a sidelying position
Head Bobbing: Subtle movement of the head due to a lack of stability
Alignment: The proper positioning of body parts in relation to each other

Kicking

Procedure: Examiner places hands on baby's legs; flexes hips and knees to chest; then releases legs.

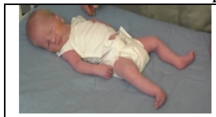	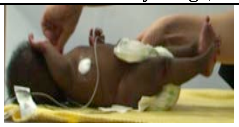	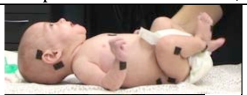 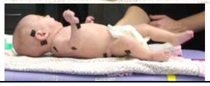	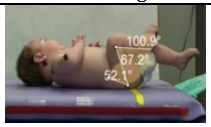
**Score 0**	**Score 1**	**Score 2**	**Score 3**
**Select score in which you observe a majority of the following:**			
• No kicking elicited<br>• LE fall passively onto support surface<br>• LE difficult to extend<br>• No flexor recoil	• Kicking elicited with limited hip flexion; primarily extension	• Unilateral or bilateral kicking elicited with sustained LE flexion for <5 seconds<br>• Delayed flexor recoil but observed in <5 seconds	• Reciprocal kicking elicited with sustained LE flexion for >5 seconds<br>• Immediate flexor recoil within 1-2 seconds after legs released
• **Any asymmetrical LE movements? ____ Yes ____ No (Side Preference? L R)**			

Head/Neck Movements in Supine without Visual Stimulation

Procedure: Infant is placed supine on mat and head is placed in midline with body then released.

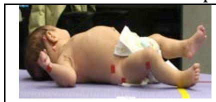	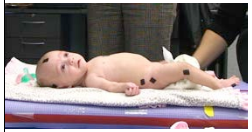 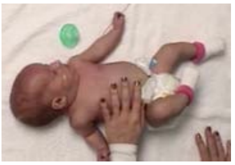	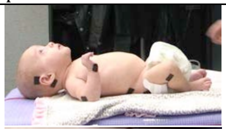 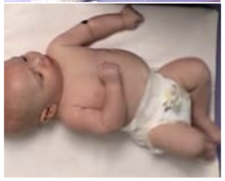	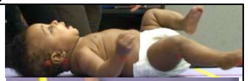 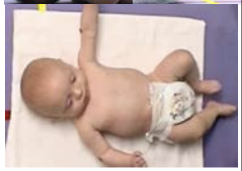
**Score 0**	**Score 1**	**Score 2**	**Score 3**
**Select score in which you observe a majority of the following:**			
• Neck and head extends or rests to side<br>Eyes Closed and does not respond, or takes >5 sec.	• Head falls immediately to side, but attempts to bring head to midline<br>• Eyes open, but eye movements jerky; does not inspect surroundings	• Infant actively inspects surroundings<br>• Able to maintain head in midline briefly; but only able **2-3 sec** then falls to the right or left	• All movements in Score 2 and<br>• keeps head in midline **≥5 secs**.

Head/Neck Movements in Supine with Visual/Auditory Stimulation

Procedure: Infant is placed supine on mat and head is placed in midline with body then released. Visual and/or auditory stimulus is an infant rattle, 8-10 inches away from face.

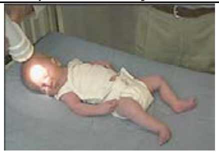 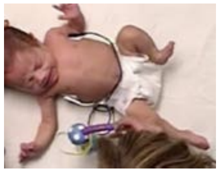	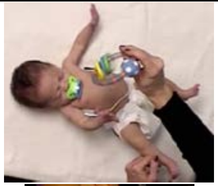 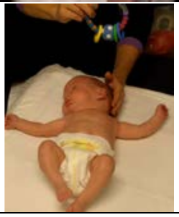	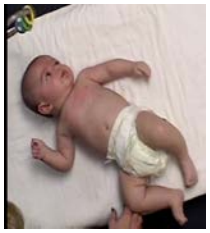	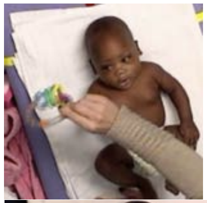 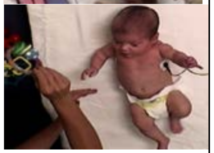
**Score 0**	**Score 1**	**Score 2**	**Score 3**
**Select score in which you observe a majority of the following:**			
• Resting posture asymmetrical<br>• Increased or decreased muscle tone<br>• **Eyes closed** and does not respond to stimuli	• Does not initially regard stimuli<br>• Responds to stimuli in **≥4 secs**.<br>• Focuses on stimuli **≥2 secs**<br>• uses whole body to move head to track stimuli	• Responds to stimuli in **<_2 secs**<br>• Can focus on stimuli **≥ 5 secs**<br>• Rotates head to track rattle, but inconsistent;<br>• Asymmetry noted	• Same posture as Score 2 and<br>• infant responds immediately to stimuli<br>• Can focus on stimuli **≥10 secs**<br>• Rotates head to consistently track rattle to both sides

Rolling Elicited by LEG

Procedure: Infant is supine on mat. Examiner places head in midline and hand on infant's upper leg and rolls infant from supine towards prone.

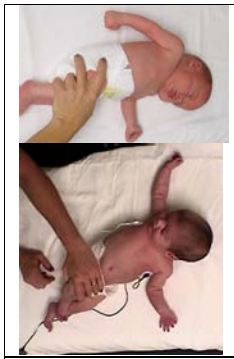	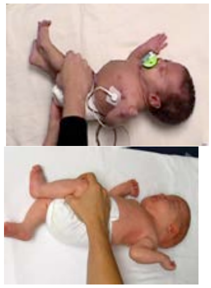	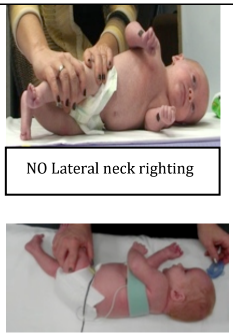	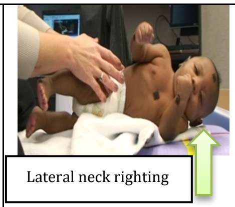
**Score 0**	**Score 1**	**Score 2**	**Score 3**
**Select score in which you observe a majority of the following:**			
• Head falls to side, slow to turn and not with trunk<br>• Arm remains inactive; stays in contact with support surface	• Excessive neck extension<br>• Unable to align head with trunk by sidelying<br>• Arm is active but lags behind trunk	• Head aligns with trunk by sidelying<br>• In sidelying, does not lift head off support surface<br>• Upper body flexion, arm aligns with trunk by sidelying	• All movements seen in Score 2 and<br>• Attempts to lift head off support surface in sidelying (lateral righting)
**Any asymmetrical head turning? ___ Yes ___ No If yes, circle which side: R L**			

Rolling Elicited by ARM

Procedure: Infant is supine on mat. Examiner places head in midline and hand on infant's upper arm and rolls infant from supine towards prone.

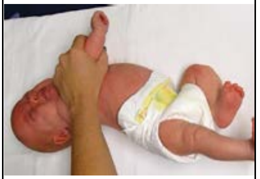	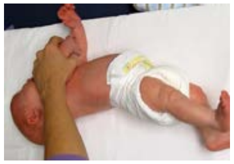	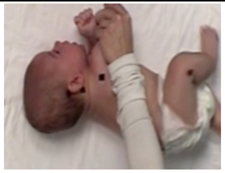	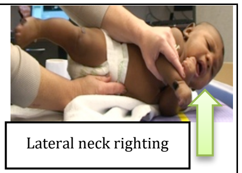
**Score 0**	**Score 1**	**Score 2**	**Score 3**
**Select score in which you observe a majority of the following:**			
• Head falls to side, slow to turn and not with trunk<br>• Leg remains inactive; stays close on in contact with support surface	• Excessive neck extension<br>• Unable to align head with trunk by sidelying<br>• Leg is active, but leg remain behind trunk	• Head aligns with trunk by sidelyng<br>• In sidelying, does not lift head off support surface<br>• Hip flexion and leg aligns with trunk by sidelying	• All movements seen in Score 2 and<br>• attempts to lift head off support surface in sidelying (lateral righting)
**Any asymmetrical head turning? ___ Yes ___ No If yes, circle which side: R L**			

.

Grasp

Procedure: Infant is supine on mat. Examiner provide deep pressure to both R and L palm and attempts to pull the infant from supine to supported sitting (traction).

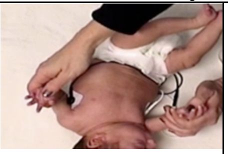	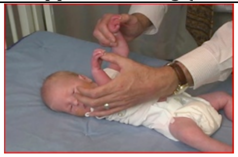	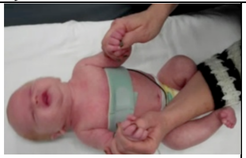	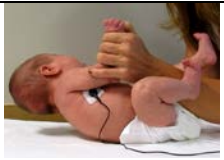
**Score 0**	**Score 1**	**Score 2**	**Score 3**
**Select score in which you observe a majority of the following:**			
• Hand is tightly fisted, difficult to open hand<br>• Fingers do not flex around stimulus<br>• Grasp is released before infant is pulled to sit.	• Incomplete flexion of all fingers<br>• Some elbow flexion with traction (pull to sit)<br>• Release of grasp during attempt to pull infant to sitting.	• Complete squeeze on examiner's finger<br>• Elbows are flexed<br>• Maintains grasp during full pull to sit	• All movements seen in Score 2 and<br>• Infant shows increased elbow flexion with traction (pull to sit)
**Any Upper Extremity Asymmetries? ___Yes ___ No If Yes, circle which limb: R L**			

Pull to Sit

Procedure: Infant is supine on mat. Examiner elicits a grasp and pulls infant into sitting.

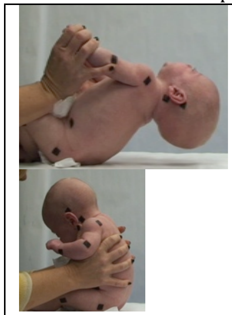	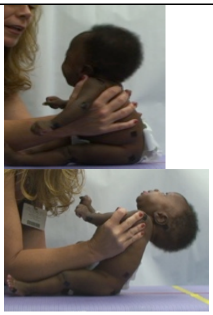	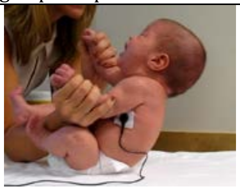	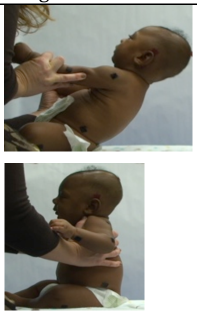
**Score 0**	**Score 1**	**Score 2**	**Score 3**
**Select score in which you observe a majority of the following:**			
• Head lag throughout pull to sit and head falls forward when up in sitting<br>• No visual awareness<br>• Knees extended	• Head lag throughout pull to sit, but keeps head at midline briefly when up in sitting; head bobbing noted<br>• Shoulder elevation during pull to sit<br>• Attempts to flex knees	• Head lag initially but able to align head to trunk by 70°<br>• Attempts eye contact with examiner<br>• May rotate head in sitting<br>• Hip and knee flexion during pull to sit	• Head lag initially, but able to align head by 45 degrees<br>• Attempts to maintain eye contact with examiner

Head Movements in Supported Sitting

Procedure: Infant is supported at trunk in upright seated position on surface; arms free to fall at sides of trunk, legs resting on surface.

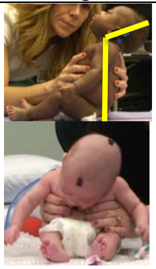	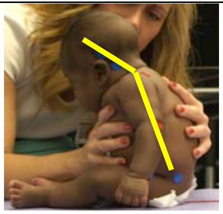	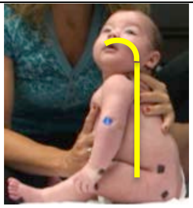	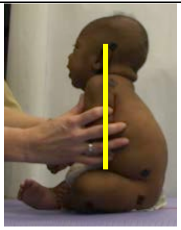
**Score 0**	**Score 1**	**Score 2**	**Score 3**
**Select score in which you observe a majority of the following:**			
• Unable to hold head at midline<br>• Head falls onto chest without attempt to pick up<br>• Infant may arch back/neck.	• Able to hold head at midline for **1 second**<br>• Makes **1 attempt** to pick head up over 10-15 seconds<br>• Head bobbing noted or head remains forward	• Able to hold head at midline **>1 to 5** seconds<br>• Makes **2 attempts** to pick up head over 10-15 seconds<br>• Tends to rotate head to side vs midline	• Able to hold head at midline **>5 seconds**<br>• Makes at least **3 attempts** to lift head to midline over 10-15 seconds
**Any asymmetrical head turning? ___ Yes ___ No If yes, circle which side: R L**			

Prone

Procedure: Infant is placed prone on mat, examiner may place head to right or left side.

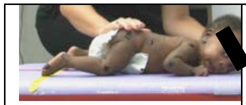	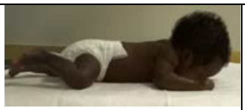	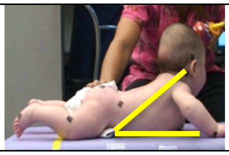	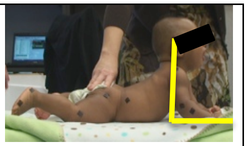
**Score 0**	**Score 1**	**Score 2**	**Score 3**
**Select score in which you observe a majority of the following:**			
• Minimal to no head lift, no rotation to side<br>• Weight shift toward head and elbows behind shoulders<br>• Hips and knees remain flexed and inactive or stiff in extension	• Attempts lift head, but, unable to reach 45°<br>• Difficulty rotating head to R and L sides<br>• Forearm movement, but elbows remain behind shoulders<br>• Hip and knee movement, but hip flexion or extension dominates	• Able to lift head to 45° head lift <5 sec<br>• May briefly lift and rotate head to both sides<br>• Forearms and elbows in front of shoulders<br>• Legs mostly kick, rarely remain still	• All movements in Score 2 and able to lift head 45°-90° head lift for at least 5 sec, emerging rotation of head<br>• Forearms bearing weight, elbow in front of shoulders<br>• Legs mostly still, stable posture

Standing

Procedure: Infant is placed in standing by examiner who supports infant around chest and arms.

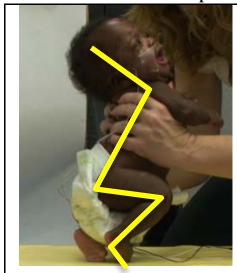	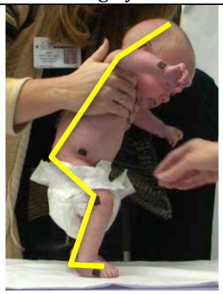	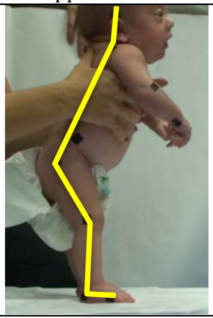	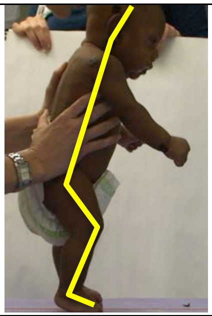
**Score 0**	**Score 1**	**Score 2**	**Score 3**
**Select score in which you observe a majority of the following:**			
• No head lifting, no alignment of head, trunk, hips.<br>• Does not take weight in legs.	• Attempts at head lifting,<br>• Able to bear weight in legs for 1-2 sec.	• Able to lift head to midline for 1-2 sec;<br>• Able to bear weight in legs for up to 5 sec..	• Able to lift head to midline > 5 sec.;<br>• Able to bear weight in legs > 5 sec.
**Any Lower Extremity Asymmetries? ___Yes ___ No If Yes, circle which limb: R L**			
