# Supplementary material for: Early developmental assessment with a short screening test, the STEP, predicts one-year outcomes
Source: J Perinatol. 2018 Oct 9;39(2):184–92. doi: 10.1038/s41372-018-0234-4 (PMC6349683; doi:10.1038/s41372-018-0234-4)
Supplement: Supplementary file 2 — Supplemental Figure 5 [file 41372_2018_234_MOESM2_ESM.pdf]

	Head Control 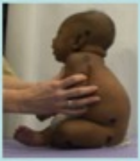	Upper Extremity 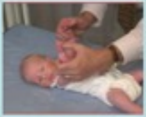	Lower Extremity 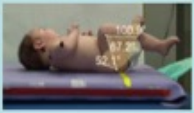
**Pull to sit**	**0.89540**	-0.23615	-0.06632
**Prone extension**	**0.89496**	-0.15936	-0.21346
**Supine with no vision**	**0.84610**	0.20399	-0.16812
**Supine with vision**	**0.76003**	-0.30853	0.16172
**Standing**	**0.69165**	-0.43411	0.23230
**Supine sitting**	**0.76553**	0.36989	0.00095
**Grasp**	0.24003	**0.72577**	-0.29594
**Kicking**	0.38154	0.48327	**0.76373**
**Rolling with leg**	**0.65745**	0.11327	-0.17037
**Percent variance explained by group**	**68%**	**20%**	**12%**
